# Multiple portal veins in the hepatoduodenal ligament: Evidence of “duodenal reverse rotation” hypothesis?

**DOI:** 10.1016/j.radcr.2023.09.027

**Published:** 2023-10-10

**Authors:** Toshihide Yamaoka

**Affiliations:** Department of Diagnostic Imaging and Interventional Radiology, Kyoto Katsura Hospital, 17 Yamada-Hirao, Nishikyo, Kyoto 615-8256, Japan

**Keywords:** Portal vein, Normal variation, Anomaly, Duplication, Prepancreatic postduodenal portal vein, Preduodenal portal vein

## Abstract

Duplication of the portal vein is a rare variation, and reports of this condition are quite limited. The present report describes a woman of advanced age who was incidentally diagnosed with duplicated portal veins. The portal vein from the splenic vein distributed to the left lobe of the liver, and that from the superior mesenteric vein ran between the pancreas and duodenum to distribute to the right lobe. The former portal vein connected with the round ligament, and its presumptive origin was the left vitelline vein. The latter was presumably from the right vitelline vein. Between the 2 portal veins, 2 anastomotic veins were identified; one anastomosis was posterior to the pancreatic head, and the other was intrahepatic. The common bile duct was located posterolateral to the portal veins. The relationships of these veins to the round ligament and common bile duct support the reverse rotation hypothesis of the duodenum in the development of portal vein variations.

## Graphical abstract

(A) Illustrated vascular anatomy and (B) embryological consideration of this case. The green line indicates the right vitelline vein and its derivative. The orange area indicates the left veins. The conventional theory of duodenal rotation cannot appropriately explain the vascular anatomy of this case.

**Figure:**
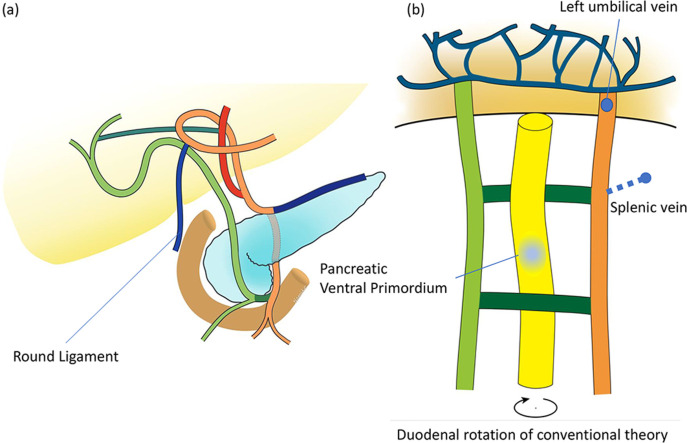

## Introduction

Duplication of the portal vein (PV) is a rare variation. However, it may result in inhomogeneous hepatic steatosis and portal hypertension. It can also be a potential barrier during surgical or radiological interventions. The number of reports of this condition has acutely increased since 2000 because of the development of imaging modalities. Knowledge of this condition can not only help to avoid inappropriate interventions but can also engage clinicians’ interests in the embryological development of the hepatoduodenal mesentery and pancreaticoduodenal complex.

## Case report

A woman in her 80s was introduced by her family doctor for evaluation of epigastric discomfort for several days. On physical examination, mild tenderness in the right upper quadrant was noted. Her laboratory tests revealed an elevated C-reactive protein concentration (6.40 mg/dL) and abnormal hepatobiliary enzyme concentrations: aspartate aminotransferase (43 U/L), alanine aminotransferase (99 U/L), alkaline phosphatase (236 U/L), leucine aminopeptidase (210 U/L), and gamma-glutamyl transpeptidase (270 U/L). She had a history of appendectomy for acute appendicitis at 19 years of age, hysterectomy and bilateral oophorectomy for uterine corpus cancer at 55 years of age, and pulmonary lobectomy for stage 1A lung cancer at 75 years of age.

**Fig. 1:**
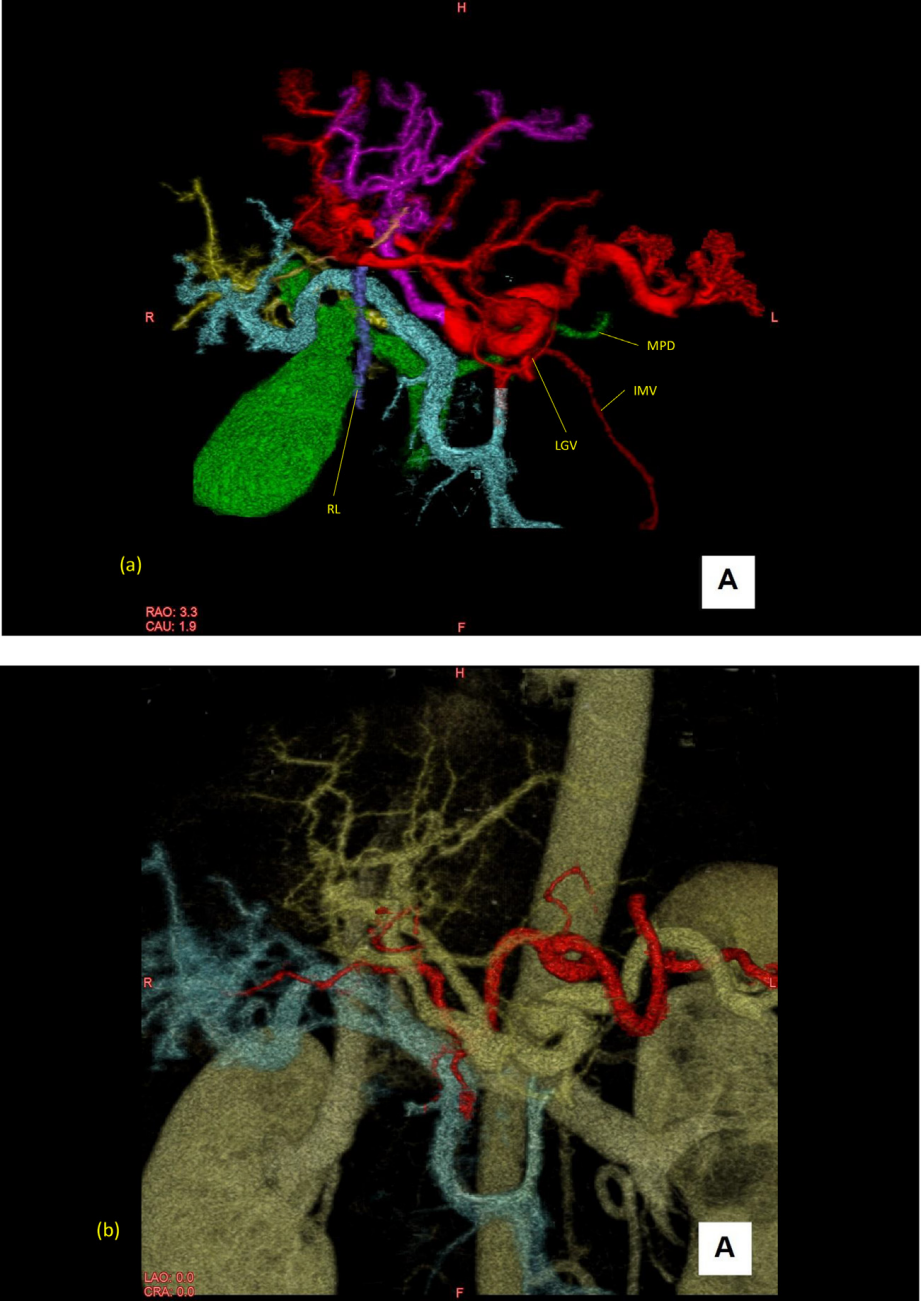
(A) Volume-rendering images of the portal veins, pancreatic duct, and common bile duct. The round ligament was located on the left side of the gallbladder; thus, the red portal vein was considered as the derivative of the left vitelline vein. (B) Volume-rendering images of the portal veins and the celiac artery and its branches. The branching pattern of the celiac artery was the most common type. RL, round ligament of the liver; LGV, left gastric vein; IMV, inferior mesenteric vein; MPD, main pancreatic duct.

Dynamic abdominal computed tomography was performed for further evaluation of the hepatobiliary system. Unenhanced computed tomography of the liver was unremarkable. Portal-phase images revealed an increased number of vascular structures in the hepatoduodenal ligament. Thin-slice data were reconstructed for volume-rendering images (Fig. 1, Supplementary Video 1).

The superior mesenteric vein was divided immediately caudal to the uncinate portion of the pancreas. The posterior branch was confluent to the splenic vein to form the PV (ie, splenic PV) behind the pancreas and ascended to the porta hepatis. The other anterior branch ran between the duodenum and pancreas and ascended to the porta hepatis, resembling a prepancreatic postduodenal PV (ie, mesenteric PV).

Both the splenic and mesenteric PVs bifurcated cephalad to the pancreas, and 4 branches of the PV ran in the hepatoduodenal ligament. The inferior mesenteric vein and left gastric vein were tributaries of the splenic PV. The pancreaticoduodenal vein joined to the mesenteric PV cephalad to the pancreas. The common bile duct was located posterolateral to the mesenteric PV and ran parallel to both PVs.

**Fig. 2:**
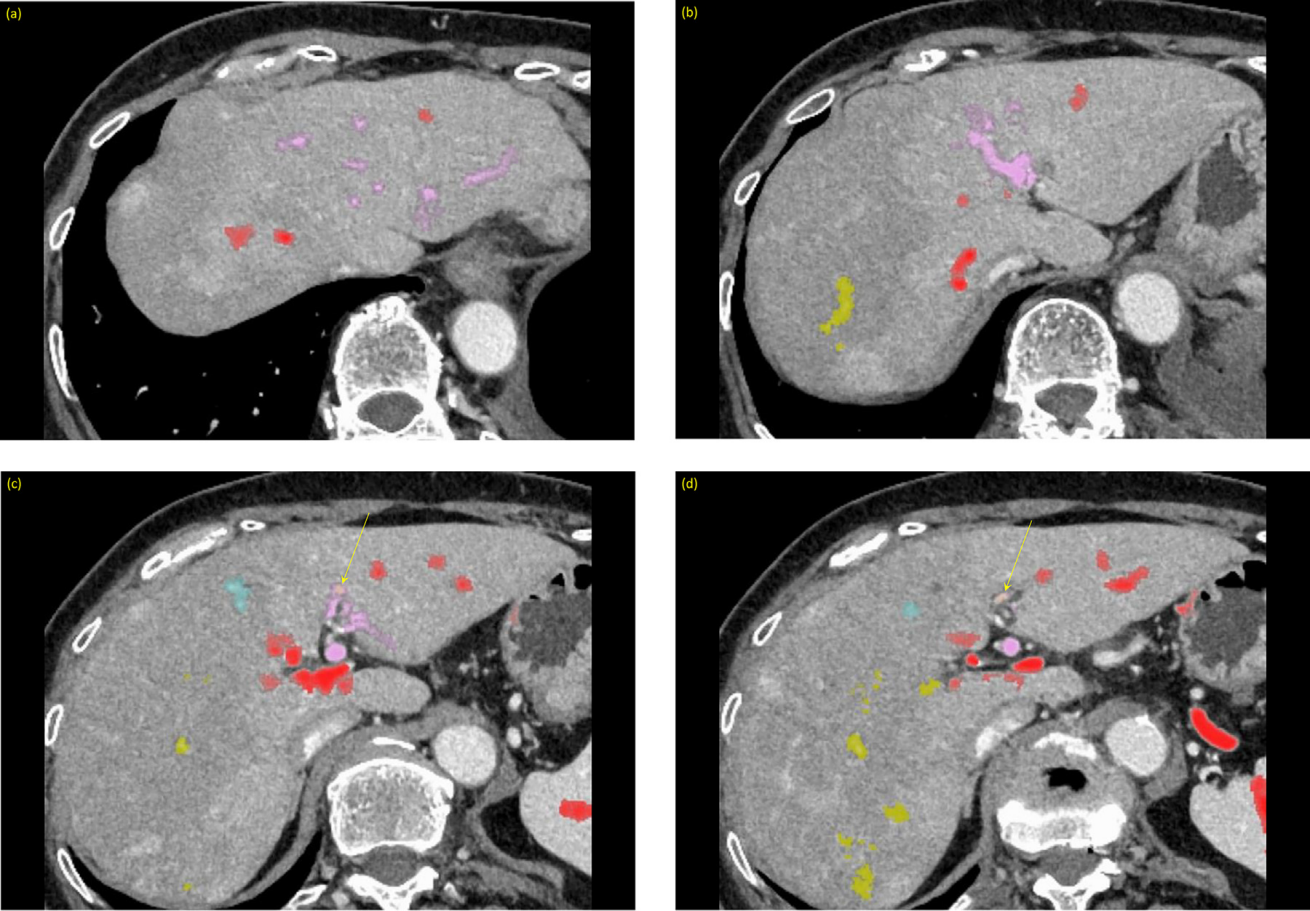

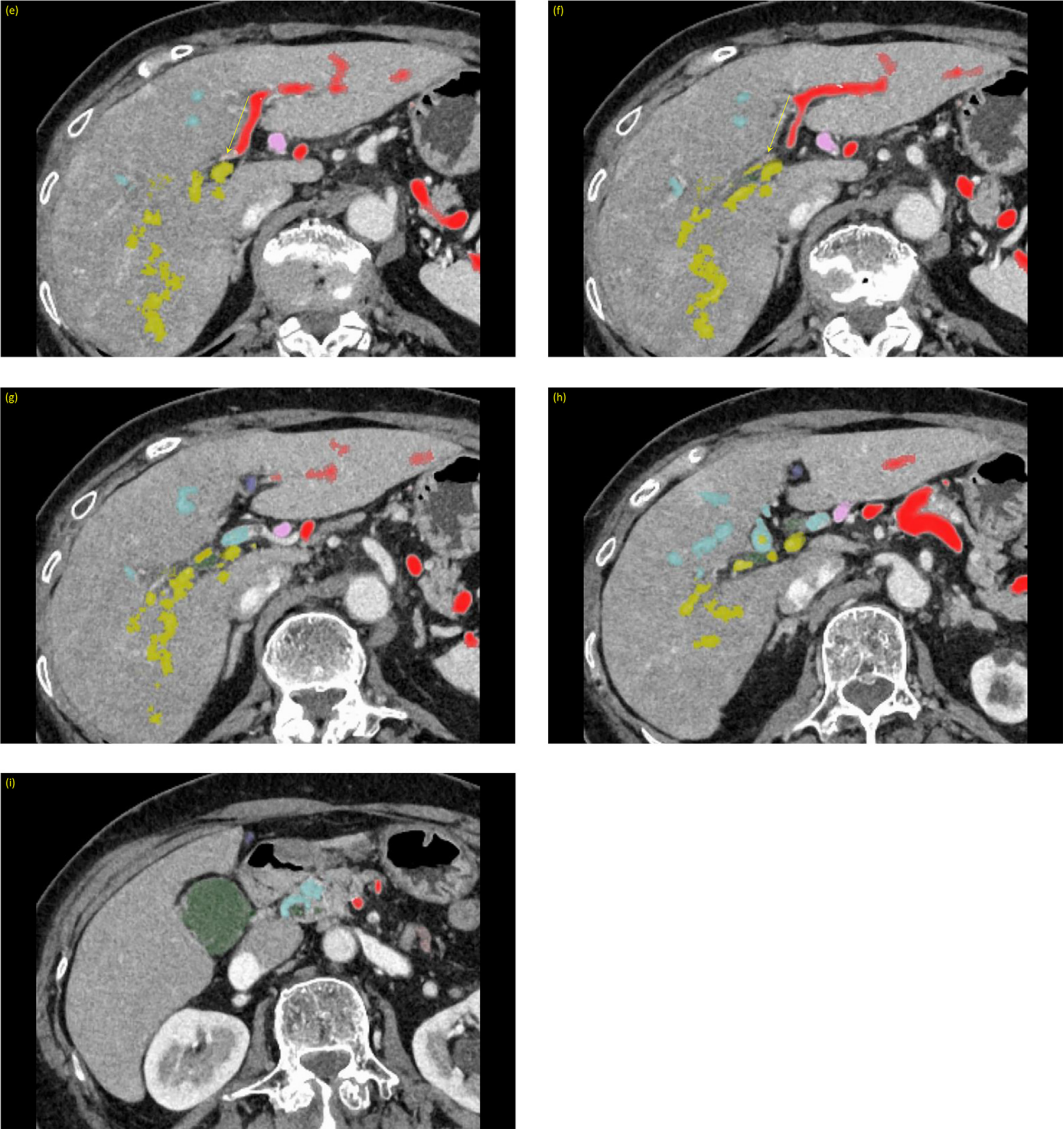
(A-I) Axial images with color mapping of each portal vein. The arrows indicate the anastomoses between the portal veins.

In the liver, the splenic PV and mesenteric PV distributed to the left and right lobes of the liver, respectively (Fig. 2, Supplementary Video 2). Anastomoses were present between the mesenteric and splenic PVs. The round ligament of the liver was connected to the splenic PV.

The patient was diagnosed with cholangitis and referred back to the family doctor for medical therapy.

## Discussion

### Literature review

The venous blood flow from the gastrointestinal tract usually feeds into the single PV passing within the hepatoduodenal mesentery and enters the liver through the porta hepatis. Venous blood flow into the liver from other pathways is called “third inflow.” In most cases, its presence is simply recognized by a focal difference in fat accumulation from other areas of the liver [1,2].

Chickens have a vein called the left PV, which runs from the stomach to the liver [3,4]. In humans, there are several case reports of a similar vessel between the stomach and liver passing through the left margin of the hepatogastric mesentery [5,6]. This vessel is considered not a left PV but an aberrant left gastric vein [1,^2^,^7^,8]. Embryologically, the human PV develops from the omphalomesenteric (vitelline) veins located on the left and right sides of the primitive duodenum. Between both vitelline veins, 3 anastomoses are thought to exist. Partial loss of these vessels results in the development of a typical human PV in the hepatoduodenal mesentery [9,10]. Therefore, it is reasonable to consider that the vessels form in the hepatoduodenal mesentery as PVs.

In addition to the cases of an aberrant left gastric vein, 12 cases of duplicated PVs have been reported [11], [12], [13], [14], [15], [16], [17], [18], [19], [20], [21], [22], [23]. Reports discussing an “accessory” PV were also found [24,25]. These reports contained various anatomical descriptions, causing conceptual confusion. A summary of the anatomical characteristics of these cases is provided in Table 1. Cases of accessory PVs are not included in this table because the anatomical relationship among the vessels, pancreas, and duodenum was unclear from the presented images.

**Table 1:** Summary of reported cases.

Green cell: PVs within the hepatoduodenal mesentery.

White cell: One PV within the hepatoduodenal mesentery, and the other PV with aberrant course.

In 10 cases including our case [11], [12], [13], [14], [15], [16], [17], [18], [19], [20], the PVs entered the liver via the porta hepatis, and they were considered a strict form of duplicated PVs. In 8 of these 10 cases, the course of the splenic and mesenteric PVs could be evaluated. Six mesenteric PVs were located ventral to the pancreas or duodenum [11,^12^,^15^,^16^,20], and only one splenic PV traversed anterior to the duodenum [11]. In the remaining 3 cases [21], [22], [23], one PV entered the liver via the porta hepatis and the other directly entered the liver parenchyma. These cases were defined as duplicated PVs in the broad sense because vessels coursing outside the hepatoduodenal ligament to the liver can be considered to provide prominent third inflow into the liver, similar to an aberrant left gastric vein.

### Embryological considerations

Among the previous reports, only Ichikawa et al. [23] reported the position of the round ligament. The position of the round ligament can be considered to indicate which vitelline vein is connected to the round ligament [10]. In other words, a left-sided round ligament connects to the left vitelline vein, and a right-sided round ligament connects to the right vitelline vein. In our case, the round ligament was left-sided and connected to the splenic PV. Therefore, the splenic PV was thought to have originated from the left vitelline vein and the mesenteric PV from the right vitelline vein.

The common bile duct was located posterolateral to the mesenteric PV. Moreover, the mesenteric PV traversed anterior to the pancreatic head, and the splenic PV traversed posterior to it. These anatomical relationships are difficult to explain by conventional rotation theory. Tomizawa et al. [9] proposed the reverse rotation hypothesis in the preduodenal PV. In our case, the mesenteric PV had a prepancreatic postduodenal course; however, its relationship to the common bile duct and splenic PV cannot be explained without the reverse rotation hypothesis.

## Conclusion

This report has described an incidentally discovered case of duplication of the PV. This case supports the reverse rotation hypothesis of the duodenum in some cases of preduodenal or prepancreatic PVs.

## Patient consent

Written informed consent was obtained from the patient.

## Appendix Supplementary materials

Image, video 1

Image, video 2

## Footnotes

Supplementary material associated with this article can be found, in the online version, at doi:10.1016/j.radcr.2023.09.027.
